# Utilization of EGFR, ALK, and BRAF Inhibitors in the Treatment of Lung Cancer in Germany

**DOI:** 10.1002/cnr2.70060

**Published:** 2024-12-18

**Authors:** Nikolaj Rischke, Josephine Kanbach, Ulrike Haug

**Affiliations:** ^1^ Department of Clinical Epidemiology Leibniz Institute for Prevention Research and Epidemiology – BIPS Bremen Germany; ^2^ Faculty of Human and Health Sciences University of Bremen Bremen Germany

**Keywords:** ALK, BRAF, claims data, EGFR, non‐small cell lung cancer, real‐world data, targeted cancer therapy

## Abstract

**Background:**

There is a lack of real‐world data on the use of targeted cancer drugs requiring molecular tumor diagnostics in the treatment of lung cancer in Germany.

**Aims:**

We aimed to characterize the use of such drugs in lung cancer patients based on longitudinal analyses.

**Methods and Results:**

Using the GePaRD database (claims data from ~20% of the German population) we identified lung cancer patients diagnosed in 2016 based on a previously developed algorithm and followed them until death, end of continuous insurance, or end of 2019. We determined the proportion of patients treated with at least one of the drugs under study (EGFR, ALK, and BRAF inhibitors). We described these patients regarding demographic characteristics, treatment patterns, and overall survival. We included 7833 incident lung cancer patients. Of these, 392 (5%) were treated with one of the drugs under study, the majority (62%) being female. In 314 out of the 392 patients (80%), the first dispensation was an EGFR inhibitor (afatinib: 54%, erlotinib: 33%), and in 72 patients (18%), it was an ALK inhibitor (crizotinib: 90%). The proportion of patients treated with these drugs was 4.8% in West Germany and 6.2% in East Germany. About half of these patients had chemotherapy before targeted therapy. Median overall survival after the first targeted therapy was 22 months.

**Conclusion:**

One twentieth of lung cancer patients diagnosed in 2016 in Germany received at least one EGFR, ALK, or BRAF inhibitor during follow‐up. The proportion was higher in East than in West Germany. As the development and availability of new cancer drugs is a dynamic area, regularly updated utilization studies—ideally as cross‐country‐comparisons—are required.

## 1 Introduction

With nearly 60 000 newly diagnosed cases per year lung cancer is one of the most common cancers in Germany. In 2018, it was the third most common cancer type in women and the second most common cancer type in men. Lung cancer has a poor prognosis and is responsible for almost 20% of all cancer deaths in Germany [1].

In recent years, many targeted drugs, especially protein kinase inhibitors, have been approved for the treatment of lung cancer, more than for any other solid tumor [2]. Unlike classic chemotherapeutic agents, these drugs target specific genes or proteins important for a cancer cell to grow or survive. Targeted therapy has significantly improved the treatment options in non‐small cell lung cancer (NSCLC). Clinical trials have shown that they offer advantages in terms of survival, and in terms of toxicity, short‐term exposure to targeted therapy drugs is generally manageable. Nonetheless, their prolonged use for chronic administration can result in significant toxic side effects [3].

Most of them are only applicable to subgroups of lung cancer patients with a specific molecular profile. To identify patients who can benefit from these therapies, molecular equipment and expertise are required, which may not always be available. In Germany, facilities for molecular tumor diagnostics of lung cancer have only been established recently, mainly at specialized and certified cancer centers [4]. Subsequently, networks have been created to ensure that patients treated at other hospitals also have access to these facilities. It is, however, not clear to which extent new cancer drugs requiring molecular tumor diagnostics are already used in the treatment of lung cancer patients in Germany. The importance of monitoring the real‐world use of these drugs has recently been demonstrated by Sadik et al. They estimated that ~64% of patients in the United States with advanced NSCLC potentially eligible for precision oncology treatments do not benefit from them because of a major clinical practice gap [5], but this may differ between countries. Furthermore, it is not clear whether there are regional differences in the utilization of these drugs.

To answer these questions, information on the utilization of new cancer drugs in the real‐world setting is required. Clinical cancer registries have been established only recently in Germany, so data on cancer treatment are not yet complete. Thus, alternative data sources are needed. Even though German claims data lack some information on cancers (histology, detailed staging), they have been shown to provide useful information on the utilization of new cancer drugs in Germany [6, 7].

We therefore aimed to characterize utilization of new cancer drugs requiring molecular tumor diagnostics for the treatment of lung cancer based on a large German claims database, exemplified by patients diagnosed in 2016 and followed up until end of 2019. Accordingly, our study refers to new cancer drugs that were already approved for NSCLC in this period (Table 1).

**TABLE 1:** Overview of the targeted cancer drugs considered in this study including corresponding ATC and OPS codes and the date of approval for non‐small cell lung cancer (NSCLC).

	ATC code	OPS code	Approved for NSCLC
EGFR inhibitors			
Erlotinib	L01EB02	—	09/2005
Gefitinib	L01EB01	—	06/2009
Afatinib	L01EB03	—	09/2013
Osimertinib	L01EB04	6‐00b.f	02/2016
Necitumumab	L01XC22	6‐009.g	02/2016
Dacomitinib	L01EB07	6‐00c.5	04/2019
ALK inhibitors			
Crizotinib	L01ED01	6‐006.c	10/2012
Ceritinib	L01ED02	6‐008.a	05/2015
Alectinib	L01ED03	6‐00a.0	02/2017
Brigatinib	L01ED04	6‐00b.3	11/2018
Lorlatinib	L01ED05	6‐00c.a	05/2019
BRAF inhibitors			
Dabrafenib	L01EC02	6‐007.5	04/2017

*Note:* Newer drugs targeting other mutations (such as the NTRK inhibitors larotrectinib and entrectinib or the RET inhibitors selpercatinib and pralsetinib) have not been examined due to insufficient follow‐up time. Likewise, the MEK inhibitor trametinib, which is only used in combination with dabrafenib, was not examined separately, as well as EGFR, ALK, and BRAF inhibitors that were approved in Germany after 2019.

## 2 Methods

### 2.1 Data Source

Our study was based on the German Pharmacoepidemiological Research Database (GePaRD) which contains health insurance claims data from four statutory health insurance (SHI) providers in Germany. The database currently includes information on approximately 25 million persons who have been insured with one of the participating SHI providers since 2004 or later. In addition to demographic data, GePaRD contains information on drug dispensations as well as outpatient (i.e., from general practitioners and specialists) and inpatient services and diagnoses. Per data year, there is information on approximately 20% of the general population and all geographical regions of Germany are represented.

Diagnoses in GePaRD are coded according to the International Classification of Diseases 10th revision, German Modification (ICD‐10‐GM). To identify lung cancer patients, we developed a sophisticated algorithm described in Figure S1. In brief, we considered patients with at least one inpatient discharge diagnosis of lung cancer (C33, C34). In addition, the algorithm takes into account that lung metastases from other cancers may partly be coded as lung cancer (as observed based on patient profile reviewing). We used a pre‐observation period of 3 years to distinguish incident from prevalent lung cancer patients. Stage at diagnosis was roughly estimated based on ICD codes indicating lymph node involvement or distant metastases as previously described [8].

In GePaRD, cancer drugs administered orally, which applies to all drugs under study here (Table 1) except for necitumumab, can be identified in the outpatient setting by their respective Anatomical Therapeutic Chemical (ATC) code. The same holds true for pre‐filled syringes. For individually prepared cancer medication administered parenterally in the outpatient setting, the active substances are available for three of the four SHIs participating in GePaRD. Inpatient dispensing of the drugs under study can be captured by specific OPS codes, irrespective of the route of application.

### 2.2 Study Population and Study Design

We included patients with incident lung cancer diagnosed in 2016. Patients with inconsistent or missing information on sex or birth year as well as patients not living in Germany were excluded. Furthermore, given that the drugs under study (Table 1) are approved for NSCLC we excluded patients receiving a therapy specific for small cell lung cancer (SCLC) (distinction based on ICD‐10 codes between both types of lung cancer is not possible). For included patients, we assigned the date of the first code for lung cancer as cohort entry and followed them up until death, end of continuous health insurance, or end of the study period (December 31, 2019), whichever occurred first.

### 2.3 Data Analysis

Our study was merely descriptive. First, we described all included patients as well as the subgroup receiving at least one of the targeted cancer drugs listed in Table 1 regarding age, sex, stage of cancer (advanced vs. non‐advanced) and location of distant metastases. We also assessed the proportion with at least one code for molecular tumor diagnostics (codes listed in Table S1). It should be noted that molecular tumor diagnostics might partly not be coded separately and the available codes are often unspecific, that is, underestimation of the proportion is to be expected. For those receiving at least one of the targeted cancer drugs under study, we described treatment patterns stratified by the class of the drug dispensed first. In terms of prescriptions or procedures, the category “missing” does not exist in claims data. If there are no codes indicating reimbursement of prescriptions or procedures, it is assumed that these have not taken place.

To assess potential regional differences in access to targeted cancer therapy, we determined the proportion of patients receiving one of the drugs under study stratified by federal state as well as 95% confidence intervals of these proportions. The federal state was assigned based on the district of residence. For those receiving a targeted cancer drug under study, we described overall survival after the first dispensation of such a drug using Kaplan–Meier analyses including 95% confidence intervals. We conducted this analysis also stratified by sex. For all analyses we used the software SAS version 9.4 (SAS Institute Inc. Cary, NC).

## 3 Results

### 3.1 Baseline Characteristics

Overall, we included 7833 lung cancer patients (Table 2). Their mean age at diagnosis was 69.0 years and 44.8% were female. The proportion assigned to the category “advanced stage at diagnosis” was 71.7%. At least one code for molecular tumor diagnostics was recorded in 30.2% of all patients. The proportion receiving at least one of the targeted cancer drugs listed in Table 1 was 5% (*n* = 392). In this subgroup, the mean age at diagnosis was 66.1 years and the proportion of female patients was 61.5%. The proportion assigned to the category “advanced stage” was 87.5% and codes for molecular tumor diagnostics were recorded in 56.9% of the patients in this subgroup. In this subgroup as well as in all included patients, the most frequent location of distant metastases was “bone/bone marrow.”

**TABLE 2:** Baseline characteristics of all included patients and the subgroup of patients receiving at least one targeted cancer drug listed in Table 1.

	All patients	Patients receiving targeted cancer drugs listed in Table 1
*N*	7833	392
Age at diagnosis (years), mean ± SD	69.0 ± 10.4	66.1 ± 11.7
Females, *n* (%)	3507 (44.8)	241 (61.5)
Males, *n* (%)	4326 (55.2)	151 (38.5)
Stage at diagnosis, *n* (%)		
Non‐advanced	2216 (28.3)	49 (12.5)
Advanced	5617 (71.7)	343 (87.5)
Location of metastases coded at diagnosis, *n* (%)		
Bone/bone marrow	1478 (18.9)	126 (32.1)
Brain	1176 (15.0)	77 (19.6)
Adrenal gland	681 (8.7)	26 (6.6)
Liver	1139 (14.5)	58 (14.8)
Mediastinum/pleura	877 (11.2)	89 (22.7)
At least one code for molecular tumor diagnostics during follow‐up, *n* (%)	2365 (30.2)	223 (56.9)

### 3.2 Treatment Patterns

In 314 (80.1%) out of the 392 patients receiving a targeted cancer drug listed in Table 1, the first dispensation was an EGFR inhibitor (Table 3). Within this group, the most commonly dispensed substance was afatinib (54.2%), followed by erlotinib (33.1%). The median time between lung cancer diagnosis and first dispensation of an EGFR inhibitor was 3.3 months. About three quarters of the patients had any cancer treatment prior to EGFR inhibitor therapy; in 45.9% of the patients, there was chemotherapy before. During follow‐up, one third of the patients received at least one other EGFR inhibitor and four patients received an ALK inhibitor.

**TABLE 3:** Characterization of treatment patterns in the subgroup of patients receiving at least one targeted cancer drug listed in Table 1.

	Patients receiving targeted cancer drugs listed in Table 1: *N* = 392		
Class of the first drug dispensed	EGFR inhibitors: *n* = 314	ALK inhibitors: *n* = 72	BRAF inhibitors: *n* = 6
Substance first dispensed, *n* (%)	Afatinib: 170 (54.1)	Crizotinib: 65 (90.3)	Dabrafenib: 6 (100.0)
	Erlotinib: 104 (33.1)	Ceritinib: 0 (0.0)	
	Gefitinib: 31 (9.9)	Alectinib: 7 (9.7)	
	Osimertinib: 6 (1.9)	Brigatinib: 0 (0.0)	
	Dacomitinib: 0 (0.0)	Lorlatinib: 0 (0.0)	
	Necitumumab: 3 (1.0)		
Age at first dispensation (years), mean ± SD	68.3 ± 10.4	60.3 ± 14.7	61.9 ± 10.4
Female sex, *n* (%)	192 (61.2)	46 (63.9)	3 (50.0)
Months between lung cancer diagnosis and first dispensation, median (interquartile range)	3.3 (1.8; 11.9)	2.9 (1.6; 14.0)	15.0 (3.7; 27.0)
Months of follow‐up, median (interquartile range)			
After lung cancer diagnosis	26.2 (14.7; 39.2)	36.8 (16.4; 40.9)	39.6 (38.0; 41.3)
After first dispensation	15.4 (5.9; 30.9)	19.3 (6.7; 36.1)	22.0 (11.9; 25.2)
Cancer therapy prior to first dispensation, *n* (%)			
Surgery	106 (33.8)	23 (31.9)	4 (66.6)
Radiotherapy	109 (34.7)	21 (29.2)	3 (50.0)
Chemotherapy	144 (45.9)	35 (48.6)	3 (50.0)
Monoclonal antibodies^a^	62 (19.7)	15 (20.8)	3 (50.0)
Any^b^	236 (75.2)	52 (72.2)	5 (83.3)
Codes for molecular tumor diagnostics before first dispensation, *n* (%)	124 (39.5)	32 (44.4)	4 (66.7)
Dispensation of another substance from the same class during follow‐up, *n* (%)	103 (32.8)	29 (40.3)	—
Dispensation of a substance from another class during follow‐up, *n* (%)	4 (1.3) (ALK inhibitor)	1 (1.4) (BRAF inhibitor)	—

^a^ Excluding the investigated targeted cancer drug necitumumab.

^b^ Including cancer therapeutics likely used for other cancer (e.g., retinoids for cancer therapy, endocrine cancer therapy), which, however, were only used in 11 of the cases with initial EGFR inhibitor therapy.

In 72 (18.4%) out of the 392 patients receiving a targeted cancer drug listed in Table 1, the first dispensation was an ALK inhibitor. Within this group, crizotinib (90.3%) was the most commonly dispensed substance, followed by alectinib (9.7%). The mean age of patients starting treatment with an ALK inhibitor (60.3 years) was 8 years lower than the mean age of those starting treatment with an EGFR inhibitor (68.3 years). The median time between lung cancer diagnosis and start of therapy with an ALK inhibitor was 2.9 months. The pattern regarding prior cancer treatment was similar to the group starting treatment with an EGFR inhibitor. During follow‐up, 40% of the patients received at least one other ALK inhibitor.

In 6 (1.5%) out of the 392 patients receiving a drug under study, the first dispensation was dabrafenib, that is, a BRAF inhibitor (used in combination with trametinib).

### 3.3 Regional Analyses

Table 4 shows the proportion of included patients receiving at least one drug under study stratified by federal state. The proportion ranged between 2.1% (Saarland) and 7.1% (Brandenburg). In the 10 federal states that were part of the former Federal Republic of Germany (“West Germany”) the overall proportion was 4.8% (95% CI 4.3–5.3). In the five federal states formerly part of the German Democratic Republic and Berlin (“East Germany”), the overall proportion was 6.2% (95% CI 5.0–7.6).

**TABLE 4:** Proportion of patients receiving at least one targeted cancer drug listed in Table 1 stratified by federal state.

	Included patients	Proportion of patients receiving targeted cancer drugs listed in Table 1
	*n*	*n* (row—%; 95% CI)
Whole of Germany	7833	392 (5.0; 4.5–5.5)
Former western part	6520	311 (4.8; 4.3–5.3)
Schleswig Holstein	408	24 (5.9; 4.0–8.6)
Hamburg	290	20 (6.9; 4.5–10.4)
Lower Saxony	895	40 (4.5; 3.3–6.0)
Bremen	295	8 (2.7; 1.4–5.3)
North Rhine‐Westphalia	1868	73 (3.9; 3.1–4.9)
Hesse	813	35 (4.3; 3.1–5.9)
Rhineland‐Palatinate	427	21 (4.9; 3.2–7.4)
Baden‐Württemberg	706	48 (6.8; 5.2–8.9)
Bavaria	723	40 (5.5; 4.1–7.5)
Saarland	95	2 (2.1; 0.6–7.4)
Former eastern part	1313	81 (6.2; 5.0–7.6)
Berlin	456	24 (5.3; 3.6–7.7)
Brandenburg	241	17 (7.1; 4.5–11.0)
Mecklenburg–Western Pomerania	176	12 (6.8; 3.9–11.5)
Saxony	167	8 (4.8; 2.5–9.2)
Saxony‐Anhalt	138	11 (8.0; 4.5–13.7)
Thuringia	135	9 (6.7; 3.6–12.2)

The proportion of patients diagnosed at an advanced stage was similar in East Germany (73.0%) and West Germany (71.4%) as well as across federal states (see Table S2).

### 3.4 Overall Survival

**FIGURE 1:**
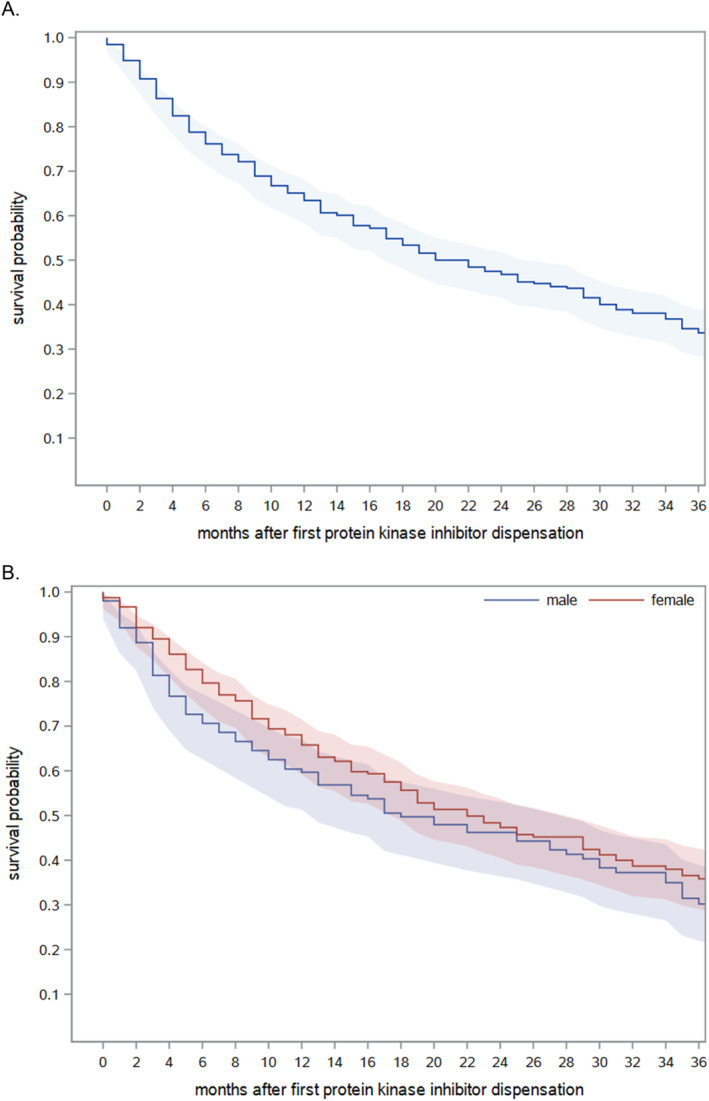
Overall survival of patients after first dispensation of a targeted cancer drug listed in Table 1. (A). All patients. (B). Stratified by sex.

**FIGURE 2:**
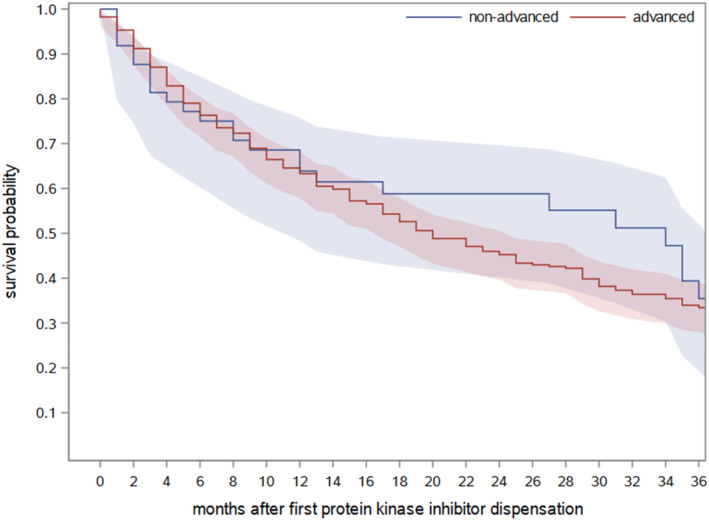
Overall survival of patients after first dispensation of a targeted cancer drug listed in Table 1 stratified by stage at initial cancer diagnosis (note that the survival curve does not start at diagnosis, but at the dispensation of a targeted therapy).

Of the 392 patients receiving a targeted cancer drug listed in Table 1, 236 (60.2%) died during follow‐up. Of those who died, about 40% (*n* = 92) died within 6 months. As shown in Figure 1A, the overall 12‐month survival probability after the first dispensation of a drug under study was 63.4% and the overall 24‐month survival probability was 46.8%. Median overall survival was 22 months (95%‐confidence interval: 17–26 months). After 24 months, there was no notable difference in survival between men and women (Figure 1B). Figure 2 shows the overall survival after the first dispensation of a drug under study stratified by stage at initial cancer diagnosis (i.e., non‐advanced means that the disease was non‐advanced at diagnosis and later progressed to a stage that was treated with a targeted therapy).

## 4 Discussion

Our study provides, for the first time, insights into the real‐world utilization of EGFR, ALK, and BRAF inhibitors in the therapy of lung cancer patients in Germany. Overall 392 (5%) out of 7833 incident lung cancer patients diagnosed in 2016 received at least one drug under study during follow‐up. In terms of regional variation, the proportion of patients treated with these drugs was higher in East Germany than in West Germany.

There is no study from Germany to which we could compare our findings. Similar real‐world studies from other countries, summarized by Spini et al. in a scoping review published in 2021, were mostly conducted in the USA and tended to focus on single inhibitor classes or specific drugs [9]. A recent study from Sweden not included in this review concluded that about 14% of advanced NSCLC patients received an EGFR inhibitor [10]. For several reasons, caution is needed when comparing this proportion to our study. First, we did not restrict our analyses to advanced NSCLC patients but included all stages. Second, also the performance status and the comorbidity, which are expected to impact on the decision whether or not to treat with targeted therapies, may be different. Third, the prevalence of specific driver mutations in NSCLC depends on ethnicity and other patient and pathological factors in addition to the histology of the tumor [11]. The prevalence reported across studies therefore strongly varies. Fois et al. estimated the prevalence of ALK aberrations in NSCLC patients to be 1%–10% according to recent epidemiological evidence but no study from Germany was available for this estimation [11]. Zhang et al. conducted a systematic review and meta‐analyses on the prevalence of EGFR mutations and reported a prevalence of 14.1% for Europe [12]. This meta‐analysis also demonstrated that smoking status and sex (apart from other factors such as race) are strongly correlated with the EGFR status, that is, patient selection in this regard matters when estimating the prevalence of EGFR. Only five studies from Germany—three with a small sample size (< 150 patients) and two with larger sample sizes (*N* = 3155, *N* = 552) but insufficient information on patient characteristics—were included in this estimate. Ideally, there would be cancer registry‐based estimates including all incident patients to estimate prevalence of these driver mutations, but such a database does not exist in Germany.

As expected, the majority (61.5%) of patients receiving one of the targeted cancer drugs under study were women. This is plausible given that the relevant driver mutations occur more frequently in females than in males [13, 14, 15]. A total of 80.1% of the patients received an EGFR inhibitor first, mainly erlotinib or afatinib. In patients who received an ALK inhibitor first (18.4%), crizotinib was the most commonly used drug. Some of the protein kinase inhibitors under study were not used as first‐line targeted therapy (e.g., dacomitinib or lorlatinib), probably because these drugs were only approved in a later phase of the observation period and at a time when other drugs of the respective inhibitor group were already available.

With respect to regional analyses, we observed a higher proportion of patients treated with new therapies in East as compared with West Germany (6.2% vs. 4.8%). We can only speculate about potential reasons for this difference. The proportion diagnosed at an advanced stage does not seem to be a plausible explanation because stage distribution was rather similar in East and West Germany. With respect to other factors, survey data showed a lower smoking prevalence in East compared with West Germany among the female cohorts from which the cases in our study originated [16]. This would mean that the share of non‐smoking lung cancer patients among all lung cancer patients is probably higher in East Germany, at least among women. Given that mutations in driver genes occur more frequently in lung cancer of non‐smokers [12], such differences could also impact on the proportion of patients receiving targeted therapies. It could also be that access to the new therapies is not the same in both parts of Germany. However, as there are more certified cancer centers in West Germany, it is not very plausible that access is worse there.

In our study, we observed a median overall survival of 22 months after dispensation of the first targeted therapy. This is lower than the median overall survival reported by pivotal RCTs on these drugs. For example, a median overall survival of 28 months was reported for afatinib [17], 23 months for erlotinib [18] and > 46 months for crizotinib [19]. When interpreting these results, it should be considered that strict exclusion criteria were applied in the pivotal RCTs, such as previous cancer therapy, active brain metastases, or a variety of comorbidities. Therefore, the discrepancy in overall survival may be due to a less selective patient selection in the real‐world setting as compared with RCTs. Similar patterns have also been observed in previous studies [6, 7, 10, 20]. Another reason for the difference in median overall survival might be the fact that targeted therapies are often used first line in RCTs, in contrast to a more heterogeneous application in the real world. The broad interquartile range of the time interval between diagnosis and first administration of a targeted therapy may also indicate that there is a mixture of first and later‐line use of these drugs, and also the approval status in this regard partly changed during the study period. The interquartile range was 1.8 to 11.9 months for EGFR inhibitors, 1.6 to 14.0 for ALK inhibitors and 3.7 to 27.0 for BRAF inhibitors. The longer intervals are partly also due to patients with lung cancer diagnosed at a non‐advanced stage (13%) that later progressed to a stage that was treated with a targeted therapy, but as shown in Figure 2, survival after dispensation of a targeted therapy seems to be rather similar in patients with advanced stage at diagnosis and those with progression after an initially non‐advanced stage.

In our study, 49 (12.5%) of the lung cancer patients treated with EGFR, ALK, or BRAF inhibitors were diagnosed at a non‐advanced stage. We assume that in several of these patients, the disease progressed to an advanced stage during follow‐up which would explain the use of targeted therapy. This is supported by the fact that half of these cases received the targeted therapy only > 12 months after lung cancer diagnosis. Given that the use of targeted therapies in early stage NSCLC is currently under debate [21], we cannot rule out that some patients may have received the drugs off label at an early cancer stage.

We found codes for molecular tumor diagnostics in 30% of incident lung cancer patients. Similar results were reported by Hardtstock et al. who also used German claims data [22]. In those receiving targeted therapy, the proportion with codes for molecular tumor diagnostics was 56.9% in our study. These relatively low proportions may indicate that mutation testing cannot fully be captured in claims data given that there is a lack of specific codes (Table S1). Also, the result of mutation testing is not available in claims data.

Our study has strengths and limitations inherent to the database we used. Specific advantages of GePaRD include the large sample size, the continuous follow‐up, and the completeness of information on new oral cancer drugs. Unlike cancer registries, the completeness does not depend on active reporting by those administering the drugs, that is, underreporting is not a concern in GePaRD. For one SHI provider participating in GePaRD, the information on the active substances in parenterally administered cancer medication dispensed in the outpatient setting is lacking. Regarding the drugs we investigated in our study, this was only relevant to necitumumab which is rarely used according to the data from the other health insurance providers, so we do not think that this had a relevant impact on our results. Unlike cancer registry data, claims data lack detailed information on stage at diagnosis and histology. This limited our possibilities to focus on NSCLC. We could only identify and exclude SCLC cases based on SCLC‐specific treatment but not all SCLC patients receive this kind of treatment. Based on the proportion of SCLC among all lung cancer cases (~15%) and the number of SCLC cases we could identify, we assume that the proportion of NSCLC cases receiving at least one drug under study would be approximately 5.7%.

It would also be interesting to investigate the impact of targeted therapy on overall survival or other effectiveness endpoints in the real‐world setting. However, this would require another type of study (causal analysis) as well as data that enable a causal interpretation, that is, data that provide all the information on patients (histology, detailed stage, performance status, etc.) that is required to control for confounding by indication or contraindication. Our study focused on drug utilization, which is highly important for identifying and monitoring potential clinical practice gaps that must be addressed through implementation research [5].

In conclusion, our study showed that one twentieth of lung cancer patients diagnosed in Germany in 2016 received at least one EGFR, ALK, or BRAF inhibitor during follow‐up. The proportion was higher in East than in West Germany. Overall survival indicated a less selective use of these drugs as compared with randomized controlled trials. As the development and availability of new cancer drugs is a dynamic area, it will be interesting and important to regularly update the study with the latest data. Furthermore, similar studies conducted in different countries based on a harmonized study protocol could provide valuable insights into potential ways to improve access to new drugs and their use in the real‐world settings.

## Author Contributions

**Nikolaj Rischke:** writing – original draft, conceptualization, formal analysis, project administration. **Josephine Kanbach:** writing – review and editing. **Ulrike Haug:** conceptualization, writing – review and editing, methodology, supervision.

## Ethics Statement

In terms of ethics approval, the Ethics Committee of the University of Bremen stated that studies based on GePaRD are exempt from institutional review board review. Apart from this, it is required to obtain approval from health insurance providers and their governmental authorities when using health insurance data for scientific purposes. The utilization of health insurance data for scientific research is regulated by the Code of Social Law in Germany. All involved health insurance providers as well as the German Federal Office for Social Security and the Senator for Health, Women and Consumer Protection in Bremen as their responsible authorities approved the use of GePaRD data for this study. According to the §75 of the Code of Social Law X, informed consent for studies based on health insurance data is required by law unless obtaining consent appears unacceptable and would bias results. For this study, the responsible authorities approved the use of the data without informed consent as informed consent was deemed to be unacceptable and to bias results.

## Conflicts of Interest

The authors declare no conflicts of interest.

## Supporting information


Data S1.

## Data Availability

It is possible to get access to the data that support the findings of this study. Please contact the corresponding author for further information. The data are not publicly available due to privacy or ethical restrictions.
