# Supplementary material for: Utilization of EGFR, ALK, and BRAF Inhibitors in the Treatment of Lung Cancer in Germany
Source: Cancer Rep (Hoboken). 2024 Dec 18;7(12):e70060. doi: 10.1002/cnr2.70060 (PMC11654761; doi:10.1002/cnr2.70060)
Supplement: Supplementary file 1 — Data S1. [file CNR2-7-e70060-s001.docx]

**Supplementary figure 1: Selection of the study population**

Patients with at least one inpatient diagnosis of lung cancer^1^ between 01/01/2016-31/12/2016 and no diagnoses indicating prevalent lung cancer in the 3 years before

N= 9,330

Patients with another type of cancer that often metastasizes to the lung

n= 1,309

n= 8,021

Patients with unknown or missing information on sex or birth year and patients not living in Germany

n= 14

n= 8,007

Specific treatment^2^ for small cell lung cancer (SCLC)
n=174

Included lung cancer cases:

n=7,833

^1^ ICD-10: C34 (Malignant neoplasm of bronchus and lung) and C33 (Malignant neoplasm of trachea)

^2^ SCLC specific treatment: cyclophosphamide, doxorubicin, epirubicin, irinotecan, lomustine, topotecan, vincristine

**Supplementary table 1: Codes indicating molecular tumor diagnostics**

| **Code** | **Description** |
| --- | --- |
| **Inpatient (OPS code)** |  |
| 1-992 | Gene mutation analysis and gene expression analysis on solid malignant neoplasms |
| **Outpatient (EBM codes)^1^** |  |
| 11211 | Basic genetic testing benefits for insured persons from the beginning of the 6th until the completion of the 59th year of life |
| 11212 | Basic genetic testing benefits for insured persons from the beginning of the 60th year of life |
| 11230 | Scientific-based human genetic assessment |
| 11301 | Basic fee for human genetic in-vitro diagnostics at the time of sample submission |
| 11320 | Detection or exclusion of a disease-relevant or disease-causing genomic mutation by hybridization with a mutation sequence-specific probe |
| 11321 | Detection or exclusion of a disease-relevant or disease-causing genomic mutation by means of sequence-specific and non-carrier-bound nucleic acid amplification |
| 11322 | Detection or exclusion of a disease-relevant or disease-causing genomic mutation by sequencing of human DNA by the Sanger chain termination method |
| 19310 | Histological or cytological examination of a material |
| 19311 | Cytological examination of a material |
| 19320 | Histological or cytological examination of a material using a special immunochemical procedure |
| 19321 | Immunohistochemical and / or immunocytochemical detection of receptors |
| 19330 | Cytological examination of a material with DNA determination |
| 19332 | Histological topography-specific determination (s) and identification (s) of the cell or tissue structure (s) to be examined on morphological examination material in connection with Fee Charges 11320, 11321 and 11322 |
| 19401 | Basic fee for tumor genetic in-vitro diagnostics |
| 19402 | Additional fee for a scientific medical assessment of complex disease-related tumor genetic analyses in an individual context |
| 19403 | Basic laboratory fee for tumor genetics |
| 19404 | Processing of a tissue or organ sample |
| 19410 | Molecular cytogenetic characterization of chromosomal aberrations at interphases or metaphases using in situ hybridization or examination for microdeletions/duplications |
| 19411 | Targeted investigation of a disease-relevant or disease-causing translocation/fusion gene |
| 19421 | Targeted detection or exclusion of a disease-relevant or disease-causing somatic genomic point mutation, deletion or duplication in coding or regulatory sequences |
| 19424 | Mutation search for the detection or exclusion of a disease-relevant or disease-causing somatic genomic mutation with clinically relevant properties |
| 19425 | Mutation search subject to approval for the detection or exclusion of a disease-relevant or disease-causing somatic genomic mutation with clinically relevant properties in more than 20 kilobases of coding sequence |
| 19450 | Molecular cytogenetic characterization of chromosomal aberrations at interphases or metaphases using in situ hybridization or examination for microdeletions/duplications |
| 19451 | Targeted investigation of a somatic genomic point mutation, a deletion or duplication in coding or regulatory sequences |
| 19452 | Targeted investigation of a disease-relevant or disease-causing translocation/fusion gene |
| 19453 | Mutation search for the detection or exclusion of a disease-relevant or disease-causing somatic genomic mutation with clinically relevant properties |
| 19454 | Search for a mutation to detect or rule out a disease-relevant or disease-causing somatic genomic mutation with clinical relevant properties in more than 20 kilobases of coding sequence including associated regulatory sequences |
| 19460 | Detection of the T790M EGFR mutation in free nucleic acids |
| 19461 | Detection or exclusion of all known EGFR-activating mutations in exons 18 to 21 using liquid biopsy |

^1^ since the EBM catalog was only supplemented with specific codes for molecular tumor diagnostics on 07/01/2016, we have included both the new precise codes (starting with 194) and the old non-specific codes also used by Hardtstock et al.

**Supplementary table 2: Proportion of lung cancer patients classified as advanced at diagnosis stratified by federal state**

|  | Included patients | |
| --- | --- | --- |
|  | n | Advanced stage,  n (row-%; 95% CI) |
| Whole of Germany | 7,833 | 5.617 (71.7; 70.7-72.7) |
| **Former western part** | **6,520** | **4,658 (71.4; 70.3-72.5)** |
| Schleswig Holstein | 408 | 283 (69.4; 64.7-73.6) |
| Hamburg | 290 | 206 (71.0; 65.6-76.0) |
| Lower Saxony | 895 | 655 (73.2; 70.2-76.0) |
| Bremen | 295 | 202 (68.5; 63.0-73.5) |
| North Rhine-Westphalia | 1,868 | 1,335 (71.5; 69.4-73.5) |
| Hesse | 813 | 570 (70.1; 66.9-73.2) |
| Rhineland-Palatinate | 427 | 302 (70.7; 66.2-74.8) |
| Baden-Württemberg | 706 | 512 (72.5; 69.1-75.7) |
| Bavaria | 723 | 529 (73.2; 69.8-76.3) |
| Saarland | 95 | 64 (67.4; 57.4-76.0) |
| **Former eastern part** | **1,313** | **959 (73.0; 70.6-75.4)** |
| Berlin | 456 | 320 (70.2; 65.8-74.2) |
| Brandenburg | 241 | 187 (77.6; 71.9-82.4) |
| Mecklenburg-Western Pomerania | 176 | 134 (76.1; 69.3-81.8) |
| Saxony | 167 | 114 (68.3; 60.9-74.8) |
| Saxony-Anhalt | 138 | 101 (73.2; 65.2-79.9) |
| Thuringia | 135 | 103 (76.3; 68.5-82.7) |
